# High-density EMG, IMU, kinetic, and kinematic open-source data for comprehensive locomotion activities

**DOI:** 10.1038/s41597-023-02679-x

**Published:** 2023-11-10

**Authors:** Hristo Dimitrov, Anthony M. J. Bull, Dario Farina

**Affiliations:** 1 Imperial College London, Department of Bioengineering, London, SW7 2AZ UK; 2 University of Cambridge, MRC Cognition and Brain Sciences Unit, Cambridge, CB2 7EF UK

**Keywords:** Biomedical engineering, Databases

## Abstract

Novel sensor technology enables new insights in the neuromechanics of human locomotion that were previously not possible. Here, we provide a dataset of high-density surface electromyography (HDsEMG) and high-resolution inertial measurement unit (IMU) signals, along with motion capture and force data for the lower limb of 10 healthy adults during multiple locomotion modes. The participants performed level-ground and slope walking, as well as stairs ascent/descent, side stepping gait, and stand-to-walk and sit-to-stand-to-walk, at multiple walking speeds. These data can be used for the development and validation of locomotion mode recognition and control algorithms for prosthetics, exoskeletons, and bipedal robots, and for motor control investigations.

## Background & Summary

The underlying neuromechanical principles of human locomotion are not yet fully understood and sensor technology plays a critical role in the studying of these processes. This knowledge gap detrimentally affects the development of control methods for prosthetics and robotics as well as our understanding of human motor control principles. Recent developments in sensing peripheral neural activity (e.g. muscle electrical activity through high-density surface electromyography - HDsEMG) or body accelerations (e.g. high-resolution inertial measurement units - IMUs), allow for obtaining a rich neuromuscular and biomechanical data to be obtained, enabling research into and analyses of human locomotion and neuromuscular control. However, currently, there are no publicly available locomotion datasets that include this advanced sensing. Datasets of this type would assist in providing new insights into basic principles of human locomotion and guide the development and testing of control algorithms and rehabilitation targets for assistive and therapeutic robotic devices. While rehabilitation robotic systems have been demonstrated to aid mobility restoration, further advances are needed, especially in relation to interfacing these systems with patients^1^. Similarly, current lower limb prostheses are limited in the restoration of natural locomotion and cause abnormal gait^2^, resulting in changes in joint and spinal loading, which may, in turn, lead to increased rates of knee and hip osteoarthritis (OA)^2–5^ and long-term spinal problems^6^. In order to improve these devices, standardised datasets with multimodal sensor recordings are needed. Furthermore, datasets of this type can be used to gain a better understanding of human locomotion in an effort to create superior models, which can help to improve the treatment and rehabilitation of human movement disorders.

Data conventionally collected during human locomotion include kinetic, kinematic, and EMG signals. While usually these data are recorded for only a few locomotion activities, it is important to provide datasets for a broad variety of locomotion modes and speeds. For example, human locomotion speed is important when characterizing healthy and pathological gait^7–9^, with gait speed directly linked to independence and quality of life in post-stroke patients^10^. Furthermore, activities such as stairs and ramps locomotion, side-stepping, and sit-to-stand-to-walk, are commonly used to evaluate gait, study human biomechanics, and develop better neuromechanical models and control algorithms for prosthetic/robotic devices.

Gait data sets including kinematics, dynamics, and, in some cases, EMG have been previously made publicly available^11–20^. However, the number of activities, recorded signals, and/or data formatting limit the application potential of many of these datasets. Some studies report only data averages^16–18^, or subject-wise averages^13^. These types of data cannot be used for training control algorithms or for investigating individual differences; they can be used for a reference comparison. Other studies have provided the raw data but lack muscle activation information (i.e. EMG)^11–15^ or have a narrow set of locomotion activities, often limited to only level-ground walking at self-selected speed or a few walking speeds^13–15,17^. A few recently published studies include EMG and have analysed different walking speeds^20^ and ambulation activities^19,21,22^. While these are very relevant datasets, there are data still missing in terms of a full spectrum of locomotion activities^20^, advanced EMG and IMU sensing^19,21,22^, and/or availability of raw motion capture data^22^. These gaps are important for data re-usability and application of novel data processing techniques (pertaining to lack of raw data) as well as capturing detailed muscle dynamics throughout locomotion (pertaining to advanced EMG sensing).

Because of these limitations of previous datasets, here we provide a comprehensive lower limb data set that includes raw motion capture marker positions, force plates data, high-resolution 6-axis IMU, 64-channel HDsEMG covering all major surface muscles below the knee of the dominant leg, and gait phase timestamps along with anthropometric data on participants’ height and weight. While our study focuses only on the dominant leg (due to wearable HDsEMG hardware limitations), the findings by Petrovic *et al*.^23^ indicate that maximal force and motor unit firing patterns are similar between the dominant and non-dominant leg, thus we believe the data from one leg are sufficient for most applications. The data was collected from 10 (5 male and 5 female) healthy able-bodied individuals during a variety of locomotion activities including: level-ground walking at slow, preferred, and fast walking speeds; stand-to-walk at preferred walking speed; sit-to-stand-to-walk at preferred walking speed; sidestepping to the left and right at preferred walking speed; stairs ascent and descent at slow, preferred, and fast walking speeds; and ramp ascent and descent using 5° and 15° ramps at slow, preferred, and fast walking speeds. This dataset can be used for the development of control algorithms for lower-limb prostheses, exoskeletons, and bipedal robots, as well as for studying and modelling bipedal locomotion and techniques for rehabilitation (e.g. model-based functional electrical stimulation).

## Methods

### Participants

This study was conducted on ten healthy participants: five males, five females, age range: 24–35 years, body height: 154–198 cm, body weight: 44.8–87.2 kg. None of the participants reported any known locomotion, neurological or other health-affecting condition that could impact their performance. Age, sex, body height and weight, as well as IMU distance from the ankle and the knee (measured from the lateral malleolus and lateral condyle respectively) of each participant were reported in the data set. The experimental evaluations were approved by the research ethics committee at Imperial College London (21IC6941) and were conducted according to the declaration of Helsinki. Each participant was briefed and provided with an information form to inform them of the procedure and potential risks before engaging in the experiment. Written informed consent was provided to all participants prior to their participation.

### Instrumentation and participant preparation

**Fig. 1:**
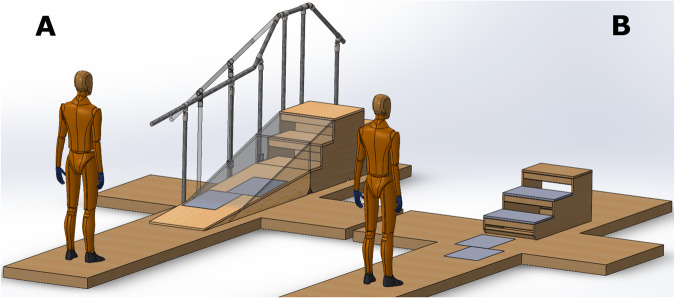
Motion capture laboratory rendering (to scale) with embedded force plates displayed in grey. (**A**) Ramp setup, where the solid ramp’s incline is 5° and the transparent one (along with the rails) is 15°. Force plates were at the same distance from the beginning of the ramp for both inclines. Force plates on the 15° ramp as well as the rails on one side are not displayed for clarity of illustration. (**B**) Level ground walking and instrumented stairs setup, where the instrumented stairs (170 mm step height, 410 mm step depth) were removed from the volume when they were not used. Human dummy model courtesy of Gil P. de Paulo^37^.

Data were collected at the Musculoskeletal Mechanics laboratory in Imperial College London. 3D kinematic motion information of the lower body was captured via a 10-camera motion capture system (Vicon, Oxford, UK) sampled at 100 Hz. Ground reaction forces were recorded using two 0.6 m × 0.4 m force platforms (Kistler, Winterthur, Switzerland) levelled with the floor, ramps, and stairs, and sampled at 1000 Hz. Motion capture lab details can be seen in Fig. 1.

A 6-axis IMU device (ICM-20602, TDK, San Jose, USA) was used to record 3D acceleration and angular velocity and a wireless 16-bit A/D EMG amplifier (Sessantaquattro, OT Bioelettronica, Turin, Italy) was used for the EMG acquisition. Raspberry Pi 4B (Raspberry Pi Ltd, Cambridge, UK) was used for the EMG and IMU data collection.

The data synchronization between the motion capture system and the Raspberry Pi was established via a square wave voltage signal, initiated at the beginning of each recording and terminated at the end of said recording.

**Fig. 2:**
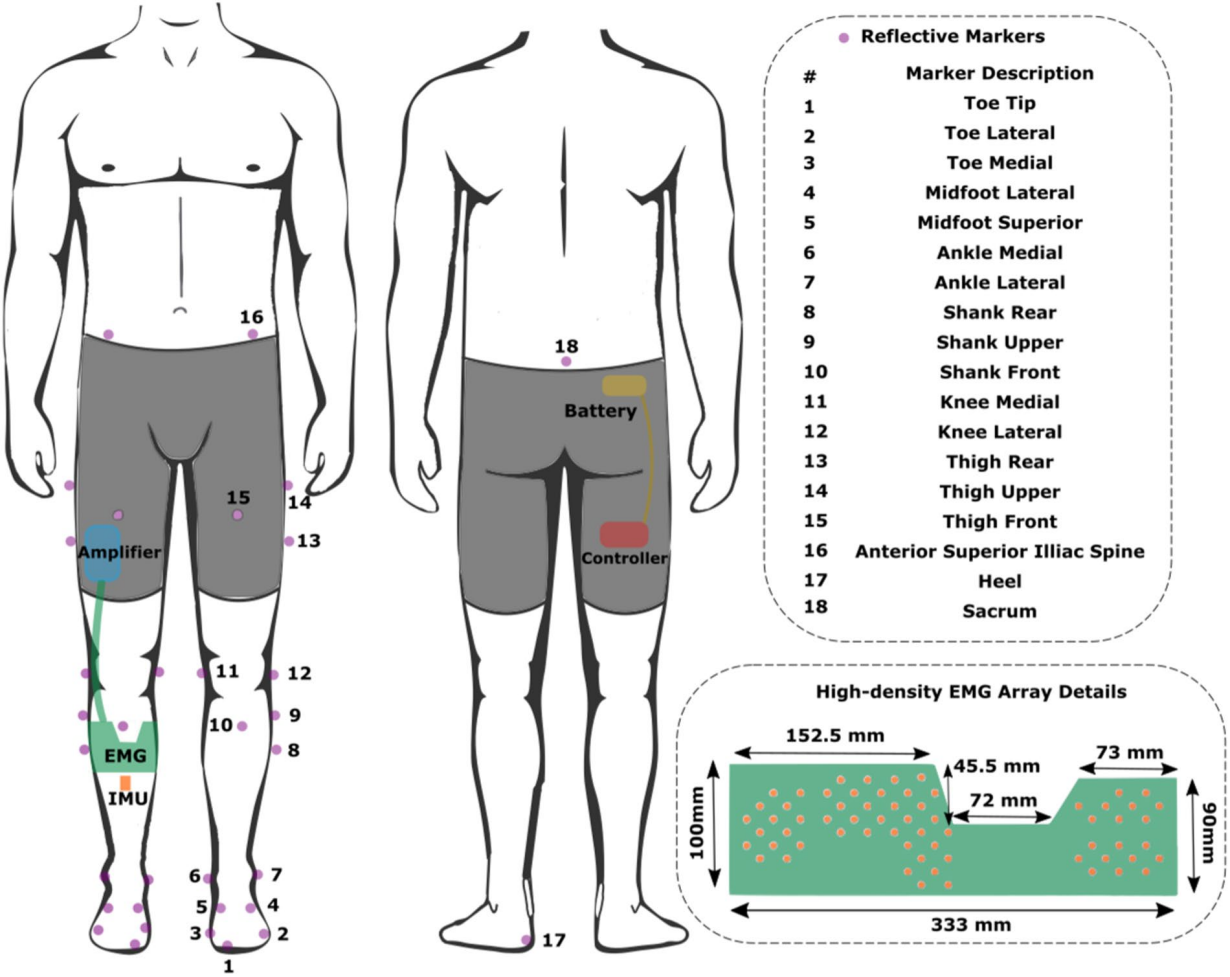
Participant instrumentation setup. Reflective markers (in purple) annotated only on the left side (apart from 18 - Sacrum) as the right side is mirrored.

Participants were required to wear a pair of custom tight thermal leggings, after which they were instrumented unilaterally (on their right side) with a custom 64-channel HDsEMG electrode grid (Fig. 2) and a 6-axis IMU sensor, both sampled at 2000 Hz and situated below the knee. The custom HDsEMG electrode grid was custom manufactured with a Kapton substrate, gold electrode contact points and copper wires. The manufacturing process was the same as the commercial grids by OT Bioelettronica^24^ (DXF CAD file for the electrode points placement provided as part of the dataset). Due to manufacturing costs and limitations of HDsEMG hardware, the grid was a one-size-fits-all. Its dimensions were derived from sampling people’s gastrocnemius length at contraction and leg circumference below the knee, assuming a relatively similar tibial width. In addition, the electrode density was determined by this area and constraints for 64 electrode contacts and was further customized to have higher density over the fibularis longus muscle, given its smaller size and significance in such recordings.

The HDsEMG grid was vertically placed right below the fibular head and horizontally aligned (using the grid’s left notch, Fig. 2) with the lateral end of the tibia. This procedure aimed to achieve consistency in placement relative to the underlying musculature, irrespective of individual variations. There will still be variations in electrode density per muscle area given the difference in participant body shapes, which is a natural limitation of electrodes that are not individually tailored to a participant, but the overall muscle activity should not be significantly dissimilar. Before placement, the area of their right lower leg, where the custom 64-channel HDsEMG electrode grid would be placed, was shaven, exfoliated, and cleaned with alcohol. Subsequently, a 6-axis IMU was placed at the centre of the tibia, right below the HDsEMG grid (distances from the knee and ankle are provided in the data structures). Following the sensor placements, the EMG amplifier and the microcontroller with its battery pack were inserted in the slots created for them in the custom thermal shorts. Finally, motion capture markers were positioned on the skin above bony landmarks, secured via double-sided tape underneath the markers and medical tape at their base, in accordance with the modified Cleveland marker placement protocol^25^. The marker set consisted of 35 retro-reflective markers (14 mm diameter) covering the lower body. An illustration of the complete participant instrumentation can be seen in Fig. 2.

### Experimental protocol

Participants engaged in two separate acquisition sessions. In the first session, they exclusively undertook level-ground walking activities on a 7-m long and 1-m wide walkway, with extra 1 m × 1.25 m platforms for sidestepping and two force plates positioned 3.15 m after the beginning of the walkway. This involved walking at varying speeds, stand-to-walk and sit-to-stand-to-walk starting with the left or the right foot first, and sidestepping to the left and to the right during walking. The second session, held separately, was solely dedicated to the ascent and descent of stairs (170-mm step height, 410-mm step depth, positioned 4.35 m after the beginning of the walkway) and ramps (5° ramp and 15° ramp, 2.3 m × 0.65 m incline path with 0.8 m plateau, positioned 4.35 m after the beginning of the walkway) at different walking speeds and ramp inclines. Each session lasted around three hours with the sensor placement and acquisition performed by the same investigator.

Before acquisition, each participant was briefed on the session details, after which they donned the custom thermal trousers used for housing the equipment. Following this, they were weighed and their height was measured, after which the equipment and markers were placed according to the procedure described in the previous sub-section. Finally, the distance from the bottom of the IMU to the lateral ankle malleolus and the lateral knee condyle was measured and recorded.

Motion capture static trial was performed at the beginning of session 1, and at the beginning of each ambulation mode (stairs, 5° ramp, and 15° ramp) for session 2. The reason for this choice was that the capture volume was kept the same but new equipment (stairs or a ramp) was introduced for the different ambulation modes; when equipment was introduced in the set-up (stairs or a ramp), the cameras were re-calibrated and a new static trial was performed. For the static trial, the participant faced forward, with arms spread at 90° to the side, and remained motionless for the short duration of the capture. After that, the participants practiced walking naturally without looking at the force plates ahead, in order to avoid changing their walking pattern due to the position of the force plates.

After calibration and walking familiarization, gait trials were performed in the various conditions. Each condition was repeated three times for a total of 27 trials for session 1 and 54 trials for session 2. For each walking condition and speed, participants had practice trials in order to ensure a natural cadence and were instructed to rest between conditions to avoid fatigue. Trials in which the participant’s foot was only partially on the force plate or on both force plates were discarded and corresponding extra trials were performed.

### Data elaboration

Following the data acquisition, the motion capture data was labelled, gap-filled (when needed), and gait events were manually identified using force plate and marker data (Vicon Nexus 2.10, Vicon, Oxford, UK). Gap filling was performed using the cyclic fill, pattern fill or rigid body fill, depending on the gap length and gap position. Heel strike and toe-off were identified as the first sample and last sample, respectively, of the force plate contacted by the relevant foot or via the vertical position of the heel and toe markers, respectively, when there was no force plate data. After this, the marker and force data were exported through the Nexus software as *.trc and *.mot files respectively and were filtered using a low-pass filter (second order zero-lag Butterworth filter, 20 Hz cut-off frequency) in MATLAB R2018a (The Mathworks Inc., Natick, MA).

The marker and force plate data were further analyzed in OpenSim^26^ using the inverse kinematics and inverse dynamics tools^27^ to determine joint angles and moments of the lower body. OpenSim’s musculoskeletal model Gait2392 was used in all the analyses. The collected static data and body mass were used to scale the model, applying scale factors and static pose weights to scale the body segments and adjust the marker positions. The scale settings used for each participant are also made available as part of the data set. Following the inverse kinematics and inverse dynamics calculations, all the data, including participant information, raw EMG and IMU, and time stamps for heel strike and foot-off, were segmented by gait cycle and arranged within a MATLAB structure. A total of 804 gait cycles for each side were analyzed.

## Data Records

All data files were acquired without any identifier and are published as such in FigShare^28^ under the terms of Attribution 4.0 International Creative Commons License (http://creativecommons.org/licenses/by/4.0/).

**Fig. 3:**
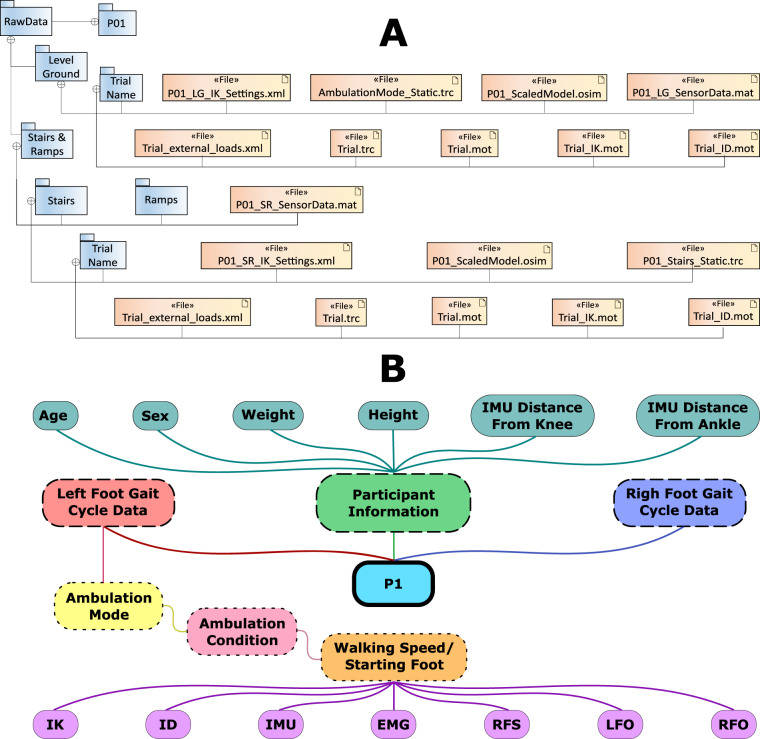
Data organization illustrating the folder structure (**A**) and the structured subject *mat data (**B**). Right foot gait cycle data has the same structure as the left one illustrated here, with the difference that instead of right foot strike (RFS), left foot-off (LFO), and right foot-off (RFO) timestamps, it contains the left foot strike (LFS), RFO, and LFO, as these are the timestamps of interest during a right foot gait cycle.

Each file is made available in *.mat format and represents a participant, with the naming convention being Pxx, where xx represents the participant number (01–10), reflecting segmented data during force plate ambulation (read below for raw data of the full trial, including transitions, Fig. 3A). Every MAT-file holds a structure, containing the respective participant metadata (Age, Sex, Weight, Height, IMU distance from the knee, and IMU distance from the ankle), left foot gait cycles, and right foot gait cycles. Left and right foot gait cycles sub-structures contain the gait cycles which start with the left or right heel strike on the force plate, respectively. For each foot, the structure is subdivided into types of walking, modes, and speeds as detailed in Fig. 3).

The data for each trial consists of raw EMG and IMU data of the right leg, inverse kinematics (IK) and dynamics (ID), time stamps for foot-offs and heel strikes during the gait cycle (in samples - same sampling rate as IK and ID, 100 Hz), as well as the respective trial name. Use a multiplication factor of 0.000286 to convert raw EMG values to mV, which is due to the wireless range and 16-bit transmission of the EMG amplifier that results in the least significant bit equal to 286 nV.

Furthermore, raw marker positions and ground reaction forces for all trials are provided in *.trc and *.mot format respectively along with complete trial IK and ID data (folder and file structure provided in Fig. 3). Raw EMG and IMU data, structured alongside labelled ambulation condition, has also been provided in a *Pxx_SensorData.mat* file for ambulation mode classification tasks.

### Data limitations

Due to the extensive recording sessions combining multiple modalities and novel sensor technology, the number and variety of participants were limited. Thus, the age range and number of participants would not allow for this data to be used as a general representation of the population. Additionally, due to the current channel limitations of wearable EMG amplifier technology, only the lower leg muscle group could be instrumented, with the aim to work and improve that in the future to span all the muscles of the lower limbs. Trials for left foot start during the stand-to-walk level ground condition for participants P01 and P04 are missing due to an investigator error.

## Technical Validation

### Motion capture and sensor data

Motion capture system calibration was performed in accordance with the manufacturer’s standard procedure at the beginning of each session, at each change of ground conditions (level ground, stairs, and different sets of ramps), as well as in cases where a camera was externally perturbed by vibration/accidental contact. Both force plates were zeroed at the beginning of the session and at each change of ambulation condition through the Vicon Nexus software.

The reflective markers and EMG array were positioned by an electrophysiology and biomechanics expert via palpation of bony landmarks and muscle tissue in accordance with the marker set guidelines and the array placement procedure described in the methods section.

Capture volume and force plate data quality were inspected at each system calibration by asking the participant to perform movements and walk along the volume. EMG and IMU signals were inspected at the beginning of the session and monitored during the trials, ensuring that the quality was consistent throughout the recording session.

### Data synchronization

Data synchronization was established via a cable between the motion capture and the sensor acquisition system. Motion capture was always initiated first, followed by the sensor data acquisition, which sends a voltage signal to the motion capture system at the start of sensor acquisition. After this, the trial began. At the end of the trial, the trigger was stopped followed by a termination of the motion capture trial. The trigger was visually inspected for every trial, ensuring the correct timing of the start and end of the trigger. Timing tests were performed before the study, to ensure the synchronization’s reliability. Furthermore, there was never a mismatch between the lengths of recordings of the acquisition systems, which were inspected after each session.

### Kinetic and kinematic data

The model used for inverse kinematics and inverse dynamics computations has been previously validated, showing average inter-examiner variability of 2.38° in joint angles and 0.33 body weight (BW) in muscle forces and a maximum of 0.33 BW variations in joint forces^29^. Another study, investigating the variability due to parameters, reported changes between 2.7° and 6.4° for joint kinematics and 2.7 to 8.1 Nm differences in joint moments^30^. These differences are consistent with other models used in the field^31–33^. The inverse kinematics and inverse dynamics results, as well as the foot events, were investigated for each trial, ensuring their correct execution. An additional check was made for the following subject-structured data, which ensured that the trials were structured and separated by gait cycle correctly.

**Fig. 4:**
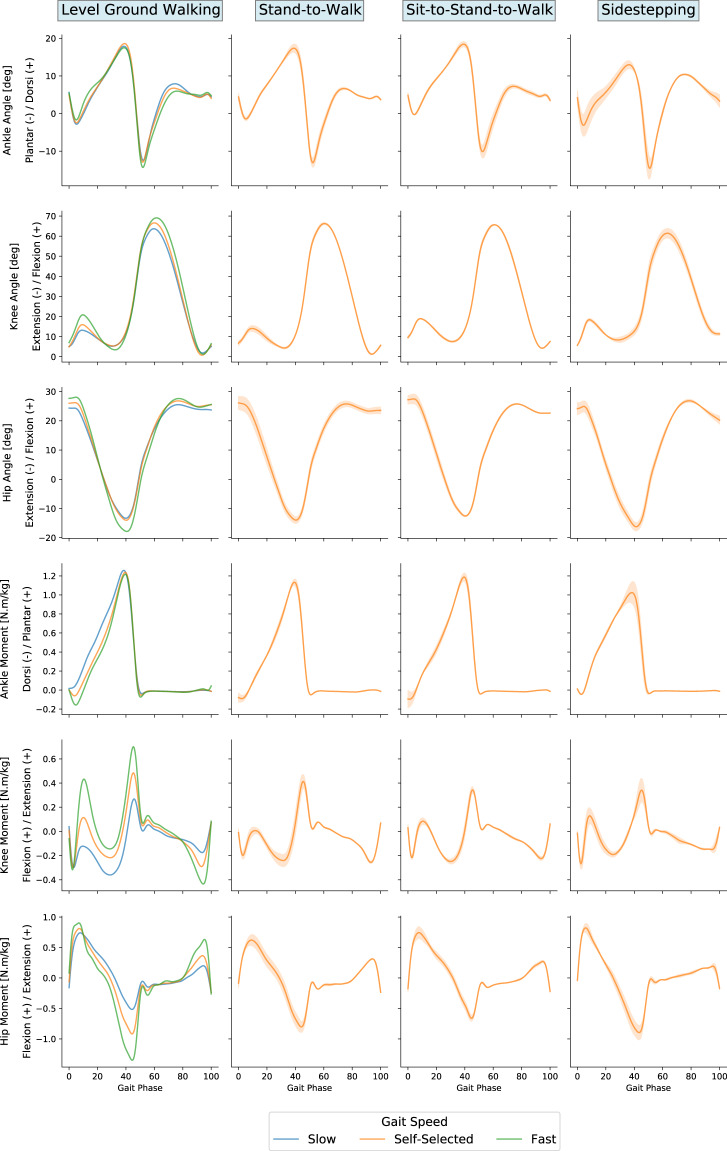
Joint angles and moments during stairs and ramps ambulation. Gait cycle duration normalized between two consecutive heel strikes of the same leg and moments normalized by the respective participant’s body weight. Tiles at the top annotate the ambulation mode.

**Fig. 5:**
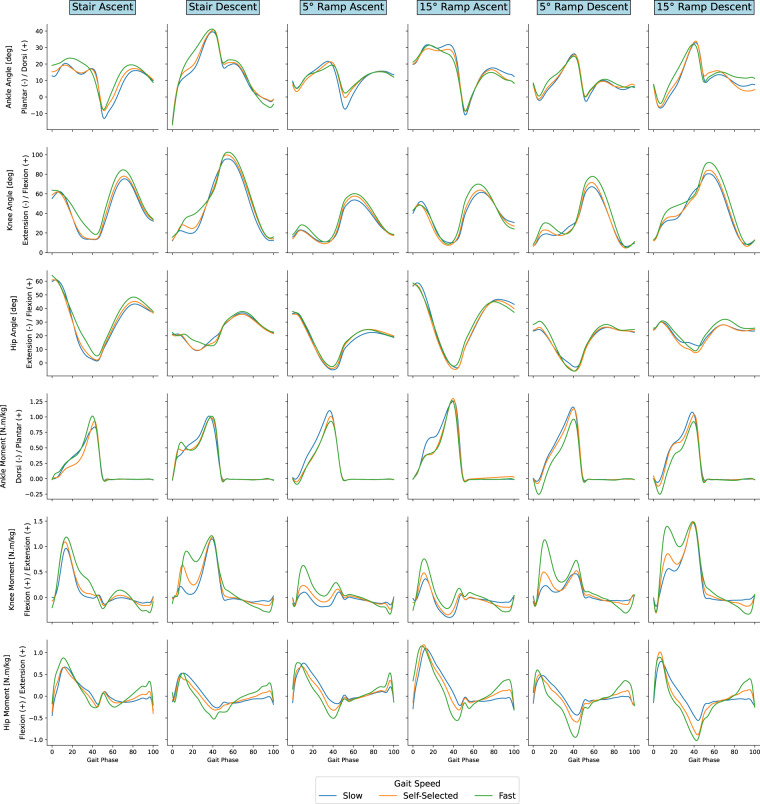
Joint angles and moments during level ground walking. Gait cycle duration normalized between two consecutive heel strikes of the same leg and moments normalized by the respective participant’s body weight. Tiles at the top annotate the walking condition. The sidestepping plots are for the foot that is in stance during sidestepping. The shaded area for the stand-to-walk, sit-to-stand-to-walk, and sidestepping represent the standard deviations for the data due to starting with the left or the right foot or sidestepping to the left or the right.

In the joint angles and moments data presented below (Figs. 4 and 5), positive values of sagittal plane angles represent ankle dorsiflexion, knee flexion, and hip flexion, and positive values of sagittal plane moments represent ankle plantarflexion, knee extension, and hip extension.

### Comparison with public walking datasets

Due to unavailability of a single data set that captures all the activities recorded in this study, the validity of this data set was assessed by comparing the angles and moments with two other public data sets that together include all the activities measured in the current study. To compare sit-to-stand along with stairs ambulation and level ground, we used the data presented by Reznick *et al*.^34^. This dataset’s population includes healthy participants of mean age of 30.4 years (±14.9), which is comparable to the population of the current data set (mean ± SD age: 27.2 ± 3.2 years). The walking speed selected for comparing level-ground data was 1.8 *m/s*, which is the closest equivalent to fast walking speed in young adults^35^ and was compared to the fast-walking speed data collected in our study. Likewise, the stair step height and ramp incline used for the comparisons were the ones closest to our setup - 6.38in step height (6.69in/17 cm step height in our setup).

To assess side-stepping along with all the rest of the activities, without sit-to-stand, our results were compared with the ones presented by Camargo *et al*. The population for this dataset is also similar to ours (mean ± SD age: 21.4 ± 1 years). Conditions that did not use walking speed variations were compared to the normal walking speed data. The ramp inclines used for comparison were 5° and 18°, which were closest to our setup (5° and 15°); and the step height used was 17.8 cm, which was the closest to our step height of 17 cm.

We computed a cross-correlation coefficient (XCOR) between the average curves of our data set and those from the selected comparison studies. The results demonstrated a high correlation between our data and those previously reported, with mean (±SD) XCOR values of: 0.98 (±0.02) and 0.82 (±0.16) for level-ground walking angles and moments respectively: 0.96 (±0.03) and 0.94 (±0.03) for stair ambulation angles and moments respectively; and 0.92 (±0.09) and 0.93 (±0.07) for ramps ambulation angles and moments respectively. Furthermore, the increase in joint torque as the walking speed increases, which was observed in the other data sets, matches our results as well.

## Usage Notes

Three MATLAB functions (combineDataStructures.m, dataInterp.m, and dataMeanPlotting.m) are provided in FigShare^28^ to assist with plotting and utilizing the data. These functions demonstrate how to combine individual participant data structures into one, interpolate gait cycles to scale them between 0 and 100, and calculate and plot data means and standard deviations for a set condition.

### Supplementary information

Human Data Checklist

## Data Availability

The code for computing joint angles and moments is provided alongside the data in FigShare^28^. The function uses the batch OpenSim processing tools^36^ to run the inverse kinematics and inverse dynamics through MATLAB and generate a folder containing “*ik*.*mot*” and “*inverse_dynamics.sto*” files for the joint angles and moments from the inverse kinematics and inverse dynamics, respectively. The code is accompanied by a README file describing these details and its usage.
